# Erfahrungen und Sichtweisen von Pflegeheimbewohnenden mit depressiver Symptomatik während der COVID-19-Pandemie: eine qualitative Studie

**DOI:** 10.1007/s00391-021-01926-3

**Published:** 2021-06-03

**Authors:** Roxana Schweighart, Malte Klemmt, Silke Neuderth, Andrea Teti

**Affiliations:** 1 Institut für Gerontologie, Universität Vechta, Driverstraße 23, 49377 Vechta, Deutschland; 2 Institut für Angewandte Sozialwissenschaften, Hochschule für angewandte Wissenschaften Würzburg-Schweinfurt, Würzburg, Deutschland

**Keywords:** Coronavirus, SARS-CoV-2, Depression, Langzeitpflege, Risikogruppe, Coronavirus, SARS-CoV-2, Depression, Long-term care, Risk group

## Abstract

**Hintergrund:**

Die COVID-19-Pandemie erfordert umfangreiche Maßnahmen in Pflegeheimen mit dem Ziel der Infektionsvermeidung. Diese Maßnahmen wirken sich auf das Leben der Bewohnenden aus und führen u. a. zu einer Zunahme an Depressivität und anderen negativen Begleiterscheinungen.

**Ziel der Arbeit:**

Die Studie hat die Exploration der Erfahrungen und Sichtweisen von Pflegeheimbewohnenden mit depressiver Symptomatik während der Pandemie zum Ziel.

**Methoden:**

Es wurden 9 leitfadengestützte Interviews mit Bewohnenden geführt, welche mittels der inhaltlich-strukturierenden Inhaltsanalyse nach Kuckartz ausgewertet wurden.

**Ergebnisse:**

Es konnten 3 Hauptthemen identifiziert werden: Empfindungen und Emotionen in Bezug auf die Pandemie, Veränderungen und Einschränkungen durch die Maßnahmen sowie Wünsche vor dem Hintergrund der Pandemie. Die befragten Bewohnenden berichten sowohl von emotionalen Belastungen als auch davon, keine Angst vor einer Infektion und deren Folgen zu haben. Zudem äußern die Befragten z. T. unangenehme Einschränkungen wie das Tragen von Masken, deren Nutzen sie bisweilen kritisch hinterfragen. Weiter werden diverse Wünsche der Bewohnenden in Bezug zur Pandemie wie etwa das Verlassen des Pflegeheims identifiziert.

**Diskussion:**

Die Studie zeigt komplexe Empfindungen, Veränderungen und Wünsche bei Pflegeheimbewohnenden aufgrund der Pandemie und der daraus resultierenden Maßnahmen. Dies erfordert einen individuellen Zugang zu den Bewohnenden mit depressiver Symptomatik, um eine Zunahme psychischer Probleme zu vermeiden. Vor diesem Hintergrund ergibt sich die Notwendigkeit einer partizipativen Umsetzung der Gesundheitsschutzmaßnahmen bei der besonders vulnerablen Gruppe der Pflegeheimbewohnenden.

## Abstract (en)

**Background:**

The COVID-19 pandemic requires extensive health protection interventions in order to prevent infections in the long-term care setting. These interventions impact residents’ lives, including an increase in depressive symptoms and other negative concomitants.

**Objective:**

The study aimed to explore the experiences and perspectives of nursing home residents with depressive symptoms during the pandemic.

**Methods:**

In this study nine guideline-based interviews were conducted with residents. These were analyzed using the content-structured content analysis according to Kuckartz.

**Results:**

Three main themes were identified: perceptions and emotions related to the pandemic, changes and limitations due to the interventions and wishes in terms of the pandemic. The residents reported both emotional distress and not being afraid of infection and its consequences. In addition, some respondents reported unpleasant restrictions, such as wearing masks. In some cases their usefulness was critically questioned. Furthermore, various wishes of the residents in relation to the pandemic, such as leaving the nursing home, were identified.

**Conclusion:**

The study showed complex perceptions, changes, and wishes due to the pandemic and its interventions. Therefore, an individual approach to residents with depressive symptoms is necessary to avoid an increase in mental health problems. Against this background, there is a need for participatory implementation of health protection measures for the particularly vulnerable group of nursing home residents.

## Hintergrund und Fragestellung

Am 28.01.2020 wurde in Deutschland die erste Infektion mit dem neuartigen Coronavirus SARS-Cov-2 (COVID-19) bestätigt. Mitte Februar empfahl das Robert Koch-Institut den Schutz besonders vulnerabler Personen, welche ein erhöhtes Risiko für schwere Krankheitsverläufe und Mortalität aufweisen, hierzu zählen u. a. Pflegeheimbewohnende [1]. Dies und die Tatsache, dass es in Pflegeheimen mit bestätigten Infektionen zu hohem Infektionsgeschehen kommt, stellt die Einrichtungen vor große Herausforderungen und führt zu diversen, z. T. restriktiven, Maßnahmen [2, 3]. Zum Schutz der Bewohnenden gelten für diese teilweise tiefgreifendere Maßnahmen als für die Allgemeinbevölkerung. Ab März 2020 bestanden massive Zugangseinschränkungen sowohl für externe Dienstleister und Leistungserbringer als auch für ehrenamtliche Helfende. Weiter wurde auch der Besuch Angehöriger temporär ausgesetzt oder nur in Ausnahmefällen erlaubt [2]. In der Alltagsgestaltung wurden tägliche Beschäftigungen und Gruppenaktivitäten wie auch die gemeinsam eingenommenen Mahlzeiten eingestellt; teilweise wurden Bewohnende in ihren Zimmern isoliert [4]. Diese Einschränkungen gehen mit negativen Folgen für die Gesundheit der Bewohnenden einher. In Frankreich und den Niederlanden, wo ähnliche Einschränkungen galten, wurden erhöhte Depressivität, Ängstlichkeit, Einsamkeit und eine Zunahme an Verhaltensauffälligkeiten festgestellt [5, 6]. Soziale Isolation und Einsamkeit können sich durch eine Vielzahl von Folgeerscheinungen (Bluthochdruck, kardiovaskuläre Erkrankungen, gesteigerte Depressivität, Abbau der kognitiven Leistungsfähigkeit) negativ auf den Gesundheitszustand von Pflegeheimbewohnenden auswirken und zu einer erhöhten Sterblichkeit führen [7–9].

Bereits vor der COVID-19-Pandemie zeigte knapp die Hälfte der Bewohnenden in deutschen Pflegeheimen Hinweise auf eine depressive Symptomatik [10].

Zielsetzung der Studie war es, die Auswirkungen der Pandemie auf das alltägliche Leben der Bewohnenden mit depressiver Symptomatik in stationären Pflegeheimen zu beleuchten und ihre subjektiven Sichtweisen und Erfahrungen zu explorieren.

## Methode

### Studiendesign

Da die Pandemie eine nie vorher dagewesene Situation in den Pflegeheimen darstellt und somit noch wenig über die Auswirkungen auf das Leben der Bewohnenden mit depressiver Symptomatik bekannt ist, wurde ein exploratives Studiendesign gewählt. Die Erfassung der subjektiven Wahrnehmungen der Befragten erfolgte über 9 leitfadengestützte Interviews. Die Durchführung der Studie und die Befundgenerierung wurden gemäß der COREQ-Checkliste [11] protokolliert.

### Sampling und Rekrutierung

In die Befragung eingeschlossen wurden Bewohnende aus stationären Pflegeheimen, bei denen eine depressive Erkrankung in der Patientenakte vermerkt war, oder die vom Personal als depressiv eingeschätzt wurden. Das Pflegepersonal wählte Personen aus, die es aufgrund seiner Fachkenntnisse als depressiv einschätzt. Das Personal führte kein Screening o. Ä. durch, sondern wählte aufgrund des täglichen Umgangs mit den Bewohnenden aus. Ausreichende kommunikative sowie kognitive Fähigkeiten stellten ein weiteres Einschlusskriterium dar. Ausgeschlossen wurden Bewohnende, die laut Patientenakte an einer fortgeschrittenen Demenz erkrankt waren. Mögliche Studienteilnehmende wurden von den Mitarbeitenden der Pflegeheime identifiziert. Potenzielle Befragte bzw. deren gesetzliche Betreuungen wurden mündlich und schriftlich über das Projekt informiert. Ein „informed consent“ wurde mittels schriftlicher Einwilligungserklärung erfasst.

### Datenerhebung

Die Erhebungen erfolgten im Dezember 2020 in 2 Pflegeheimen in Würzburg unter situationsangepassten Bedingungen wie dem Tragen einer FFP2-Maske und ausreichendem Abstand. Zum Interviewzeitpunkt lag ein negativer PCR-Test der Interviewerin vor. Die Interviews wurden in den privaten Zimmern der Bewohnenden geführt; die Interviewdauer betrug im Mittel 43 min (Min. = 30, Max. = 74). Zur Standardisierung der Interviews wurde ein Leitfaden eingesetzt, welcher folgende Leitfragen umfasst: (1) Wie geht es Ihnen seit Beginn der Pandemie? (2) Wie hat sich Ihr Leben seit Beginn der Pandemie verändert? (3) Haben sich Ihre Wünsche und Bedürfnisse durch die Pandemie verändert? Wenn ja, wie sah diese Veränderung aus? (4) Was war für Sie hilfreich und unterstützend während der Pandemie? (5) Was wünschen Sie sich für die Zukunft? Ergänzend wurden (6) soziodemografische Variablen erfasst und (7) ein Depressionsscreening anhand der 10 Items umfassenden Skala „Depression im Alter – Skala“ (DIA-S) [12] durchgeführt.

### Datenanalyse

Die Interviews wurden auf Tonband aufgezeichnet und nach feststehenden Regeln transkribiert [13]. Die Auswertung des Datenmaterials (Interviewtranskripte) erfolgte nach der inhaltlich-strukturierenden Inhaltsanalyse nach Kuckartz deduktiv-induktiv [14] und durch 2 Personen unabhängig voneinander nach dem Verfahren des konsensuellen Kodierens [15].

Mit dem finalen Kategoriensystem wurde das gesamte Datenmaterial codiert. Die Auswertung erfolgte unter Nutzung der Software MAXQDA 2020 [16].

## Ergebnisse

### Stichprobe

Insgesamt wurden 10 Bewohnende befragt. Das Interview mit einer Bewohnerin (B2) wurde nach 5 min aufgrund der gesundheitlichen Verfassung abgebrochen und nicht in die Auswertung einbezogen. Die finale Stichprobe besteht aus 9 Befragten (weiblich: *n* = 5). Die Bewohnenden waren zum Zeitpunkt der Interviews im Schnitt 81,9 Jahre alt (SD = 7,8; Range = 71–93) und lebten zwischen einem Monat und mehr als 4 Jahren im Pflegeheim. Bei 5 Befragten war eine Erkrankung aus dem depressiven Formenkreis in ihrer Patientenakte dokumentiert, während 4 Bewohnende aufgrund der Einschätzung des Pflegeheimpersonals eingeschlossen wurden. Die Schwere der Depressivität lag im Mittel bei 5,9 Punkten auf der DIA‑S. Der Wert war bei den Bewohnenden mit dokumentierter Diagnose (M = 5,8) etwas geringer als bei den vom Personal als depressiv eingeschätzten Bewohnenden (M = 6,0). Eine als sehr wahrscheinlich geltende Depression lag laut DIA‑S bei allen Befragten vor. Weitere Merkmale der Stichprobe sind Tab. 1 zu entnehmen.

**Table 1:** 

Kürzel	Geschlecht^a^	Alter^b^	Dokumentierte Depressionsdiagnose^c^	Familienstand, Kinder	Länge des Aufenthalts^d^	Punkte DIA‑S^e^
B1	M	71	Schwere depressive Episode mit psychotischen Symptomen	Verwitwet, 0	53	4
B3	W	75	Depression	Verwitwet, 1	9	8
B4	W	83	Depression	Verwitwet, 2	23	4
B5	W	90	Schwere depressive Episode	Verwitwet, 2	2	4
B6	W	73	Depression	Geschieden, 3	30	9
B7	M	76	N.v.^f^	Verwitwet, 0	6	5
B8	M	93	N.v.	Verwitwet, 1	36	6
B9	W	90	N.v.	Verwitwet, 0	9	5
B10	M	86	N.v.	Ledig, 0	1	8

^a^W = weiblich; M = männlich

^b^ In Jahren

^c^ Wie vermerkt in Patientenakte

^d^ Zeitpunkt vom Einzug bis zum Interview in Monaten

^e^ 0 bis 2 Punkte: unauffällig; ab 3 Punkten: Depressionsverdacht; ab 4 Punkten: eine Depression von Krankheitswert ist wahrscheinlich

^f^ n.v.: nicht vorhanden

### Empfindungen und Emotionen in Bezug auf die Pandemie

Die Bewohnenden äußern durch die Pandemie und die Existenz von SARS-CoV‑2 keine oder nur wenige psychische Belastung zu empfinden. Mitunter berichten sie, dass sie die Pandemiesituation als nicht schlimm oder angsteinflößend erleben und sich emotional nicht viel verändert habe.

Das [die Pandemie] war nicht schlimm für mich. Ich bin zurechtgekommen. (B1)

Die Bewohnenden zeigen wenig Furcht vor einer möglichen Infektion mit dem Coronavirus und erklären dies damit, dass sie ihr Leben gelebt haben, keine Angst vor dem Tod haben und sowieso nichts ändern können, sollte es zu einer Ansteckung kommen.

Angst nicht, das kann man nicht sagen, man muss es hinnehmen, wie es kommt […] da habe ich keine Bange vor. Da kann ich nur sagen, wenn es mich erwischt, dann muss ich halt durch die Tür gehen. Wobei ich auch keine Angst vor dem Sterben habe. (B7)

Ich bin kein Mensch, der ängstlich ist, einmal. Und sagen wir, wenn ich Unglück habe, dann habe ich Pech, nehme ich was auf oder nicht. (B8)

Es werden jedoch auch negative Auswirkungen der Pandemie auf die Empfindungen und Emotionen genannt. Zwei Personen erzählen von ihren erlebten Sorgen und Ängsten, eine mögliche Infektion oder einen Infektionsausbruch im Pflegeheim betreffend.

Es ist ja so, dass die Leute ja hier rausdürfen, ja? Sie können ja ohne Weiteres raus, und da habe ich schon irgendwie Bedenken, wie draußen, ob sie wirklich eine Maske tragen oder nicht. Also, da muss ich schon sagen, dass ich da ein bisschen Angst habe. (B5)

Eine Bewohnende berichtet von Sorgen um ihre Tochter und einer damit verbundenen emotionalen Last.

Das Corona, muss ich ganz ehrlich sagen, macht mir manchmal insofern Angst, nicht, was mich betrifft, sondern meine Tochter. Da mache ich mir Sorgen. (B3)

Eine Bewohnende äußert, sich generell belasteter zu fühlen, während eine weitere Befragte erzählt, dass sie sich einsamer fühle und sich die bereits vor der Pandemie erlebte Angst verschlimmert habe.

B6: „Angst habe ich ja sowieso, aber das ist psychisch.“

I: „Ist das stärker geworden durch Corona?“

B6: „Schwerer ist es schon geworden, ja.“

### Veränderungen und Einschränkungen durch die Maßnahmen

Die Befragten berichten von vielfältigen und zahlreichen Veränderungen und Einschränkungen in ihrer täglichen Lebensführung. Diese betreffen u. a. Restriktionen in Bezug auf das Verlassen des Pflegeheims und teilweise auch des Zimmers.

Wir waren im März wie das war, haben wir im Zimmer bleiben müssen und das waren fünf Monate (..) dann haben wir erst wieder raus gekonnt. Aber im Freien, zum Fortgehen, zum Einkaufen, da war es nichts. (B1)

Auch das eingeschränkte Besuchsrecht ist v. a. für Bewohnende mit regelmäßigem Kontakt zu Angehörigen eine große Veränderung. So berichten die Befragten z. T. von einem kompletten Besuchsverbot und teils von der Möglichkeit, die Angehörigen begrenzt sehen zu können.

Verändert hat sich also, dass zum Beispiel meine Kinder nicht mehr so oft kommen dürfen. Die dürfen ja bloß einmal in der Woche kommen, und das nur abwechselnd einen Tag. Und jetzt zum Beispiel Weihnachten. Voriges Jahr waren wir halt allerweil nachmittags da […] und haben Kaffee getrunken, dürfen wir ja auch nicht mehr. (B4)

Auch das Fehlen von Beschäftigungen und Aktivitäten wird von den Befragten als deutlicher Einschnitt in ihrem Leben wahrgenommen. Die Bewohnenden berichten von Langeweile und Untätigkeit. Weiter werden folgende Maßnahmen bei der Frage nach Veränderungen genannt: regelmäßiges Lüften und dadurch Frieren im Winter, eingeschränkter Kontakt zu anderen Bewohnenden sowie geltende Hygienemaßnahmen. Unter diesen Maßnahmen wird besonders das Tragen von Masken als lästig empfunden. So berichten die Befragten, dass die Masken unangenehm zu tragen wären, sie damit nur schlecht Luft bekommen, darunter stark schwitzen, die Brille ständig anlaufe und das gleichzeitige Tragen von Hörgerät, Brille und Maske schwierig sei.

Ich habe das jetzt dauernd aufgehabt und habe geschwitzt, meine Brille ist angelaufen. Durfte das nirgends runtertun. (B6)

Die Bewohnenden betrachten die Maßnahmen zur Viruseindämmung in den Pflegeheimen teils kritisch und hinterfragen diese.

Wenn meine jungen Leute kommen, dann dürfen sie, glaube ich, nur eine Stunde noch da sein. Ist allerdings meiner Meinung nach, ich kann einen Menschen innerhalb einer Stunde anstecken, ja? Und zwar ganz gewaltig und innerhalb von fünf Stunden, also wie gesagt, da hat man immer ein bisschen Zweifel, ob das so nutzvoll ist. (B8)

Ja, also viel Theater gemacht worden. Das dürft ihr nicht und das dürft ihr nicht. (B6)

### Wünsche in Bezug auf die Pandemie

Die Befragten nennen verschiedene Wünsche, die in direktem Zusammenhang mit der Pandemie und den viruseindämmenden Maßnahmen stehen. So wird beispielsweise der Wunsch nach Besserung der Situation genannt.

Ich wünsche, dass es mit Corona besser wird. Dass dann vielleicht wirklich mal dieser Impfstoff kommt, und dass es vielleicht bis nächstes Jahr im Sommer besser wird. (B4)

Des Weiteren werden auch Wünsche genannt, die direkten Bezug auf die vor Ort geltenden Restriktionen nehmen. So wünschen sich die Befragten, das Pflegeheim wieder regelmäßig verlassen zu dürfen, die Wiederaufnahme der Beschäftigungen und Aktivitäten sowie eine Lockerung der Besuchsregeln.

Das Einzige, ich wollt einmal richtig laufen […] eine große Runde. (B1)

Dass an Heiligabend meine Kinder da sind, dass wir wieder einen Kaffee trinken können miteinander. (B4)

Zwei Bewohnende berichten, dass ihnen nahestehende Personen während der Pandemie verstorben seien. Ein Verlassen des Pflegeheims war in beiden Fällen aufgrund der Verfügungen nicht möglich. Beide Bewohner wünschten sich den Besuch der Beerdigungen und sind traurig darüber, dass dies nicht möglich war.

Morgen wird der Mann beerdigt, ich wäre ja gern dort, aber es geht ja nicht. (B10)

## Diskussion

Die Studie hat zum Ziel, die Auswirkungen der Pandemie auf das Leben von Pflegeheimbewohnenden mit depressiver Symptomatik sowie deren Sichtweisen und Erfahrungen zu explorieren.

Die Identifizierung einzelner negativer Empfindungen und Emotionen wie Ängstlichkeit, Einsamkeit oder Sorge steht in Konsistenz mit anderen Studien [5, 6]. Überwiegend berichten die Befragten jedoch, die Pandemie als wenig belastend zu empfinden und keine Angst vor einer Infektion und deren möglichen Folgen zu haben. Obwohl die Befragten gut über die Gefahren des Virus informiert zu sein scheinen und über dramatische Ausbrüche in nahegelegenen Pflegeheimen Bescheid wissen [17], erwecken sie stellenweise einen sorglosen Eindruck angesichts einer potenziell letalen Erkrankung. Eine Erklärung hierfür könnten die Charakteristika der Bewohnenden sein. Höheres Alter, Leben im Pflegeheim und Depression können in Zusammenhang mit einem Todeswunsch, wie ihn ein Befragter mitunter kommuniziert hat, stehen [18]. Dies könnte zu Akzeptanz der eigenen Sterblichkeit und zu Sorglosigkeit gegenüber SARS-CoV‑2 führen.

Eine Studie [19] zeigt, dass ältere Menschen, die zu Hause leben, weniger durch die Pandemie belastet sind als jüngere Personen. Das lässt auf Bewältigungsstrategien schließen, die mit Erreichen eines gewissen Alters assoziiert sind und die Sorglosigkeit ebenfalls erklären könnten.

Obwohl zum Zeitpunkt der Interviewdurchführung z. T. noch erhebliche Einschränkungen in den untersuchten Heimen galten (keine Gruppenaktivitäten, wöchentlicher Besuch auf eine Person und Stunde beschränkt, dauerhaftes Tragen einer FFP2-Maske außerhalb des Zimmers etc.), waren die Reglementierungen aufgrund umgesetzter Hygienekonzepte und einer Ausweitung der Teststrategien lockerer als noch zu Beginn der Pandemie. Beispielhaft ist hier zu nennen, dass den Bewohnenden im Frühjahr und im Sommer ein Verlassen des Heims und teilweise auch des Zimmers nicht gestattet war. Im Dezember, zum Erhebungszeitpunkt, waren diese Einschränkungen aufgehoben. Lockerungen im Pandemieverlauf könnten ebenfalls zu einer Entlastung bei den Bewohnenden führen, welche sie entsprechend in den Interviews benennen.

Die Befragten empfinden die geltenden Maßnahmen als lästig und störend. Mitunter wird auch deren Sinnhaftigkeit hinterfragt. Das Zentrum für Qualität in der Pflege [20] und die Deutsche Gesellschaft für Pflegewissenschaft [21] warnen davor, den Infektionsschutz über den Erhalt der Lebensqualität zu setzen und werben für eine Abwägung zwischen Viruseindämmung, der Wahrung individueller Bedürfnisse und dem Schutz der psychischen und sozialen Gesundheit. So berichten auch die Befragten von Wünschen, welche sich aufgrund der Pandemieeinschränkungen nicht realisieren lassen. Eine Nicht-Erfüllung kann sich negativ auf die Lebensqualität und die psychische Verfassung der Bewohnenden auswirken. Es ist ein schmaler Grat zwischen der Vermeidung von Infektionen und Sterbefällen und dem Versuch, die Lebensqualität der Menschen zu erhalten und damit eine Zunahme von psychischen Problemen zu vermeiden [3, 5, 6, 22].

In Situationen, in denen persönlicher Kontakt nicht möglich ist, kann die Nutzung von Alternativen hilfreich sein. So führen regelmäßige Videokonferenzen mit Angehörigen zu weniger Einsamkeit und Depressivität sowie zu vermehrter emotionaler Unterstützung [23].

Der geringere DIA-S-Wert der Bewohnenden mit dokumentierter, teils schwerer, Depressionsdiagnose könnte auf eine antidepressive Pharmakotherapie zurückzuführen sein, die die Bewohnenden mit ärztlicher Diagnose mit höherer Wahrscheinlichkeit erhalten, als Bewohnende ohne dokumentierte Diagnose. Da die Medikation der Befragten nicht erfasst wurde, können hierzu keine Aussagen getroffen werden. Darüber hinaus könnte die Wahrnehmung depressiver Symptome durch Pflegende anders sein, als bei einer ärztlichen Diagnostik oder in der Situation, in der die DIA-S eingesetzt wurde. Dies könnte die unterschiedlichen DIA-S-Werte ebenfalls erklären.

Limitationen der Studie betreffen die Selektion der Befragten, welche durch die Mitarbeitenden der Heime vorgenommen wurde. Zum einen, da der Zugang zu den Heimen aufgrund der Pandemie stark reglementiert war, zum anderen schien es mit Blick auf mögliche Infektionsrisiken sinnvoll, die Heime nur für die Interviews zu betreten. Weiter führte die Angst vor einer Infektion evtl. zu einer Zurückhaltung ängstlicher und vorsichtiger Bewohnender mit der Folge vermehrt sorgloser Teilnehmender. Auch das Tragen von FFP2-Masken und damit verbundene potenzielle Beeinträchtigungen, u. a. das Beschlagen der Brille sowie das Nicht-Erfassen der Mimik, können zu Einschränkungen in der Interviewsituation geführt haben. Drei der 9 Befragten sind während der Pandemie ins Pflegeheim gezogen. Es kann sich eine Limitation der Ergebnisse ergeben, da die Bewohnenden keinen Vergleich zu einem Leben im Heim unter nichtpandemischen Bedingungen anstellen können. Darüber hinaus besteht die Möglichkeit, dass die Depressivität im Sinne einer Anpassungsstörung aufgrund des Umzugs aufgetreten ist oder durch diesen verstärkt wurde [24].

Es wurde nicht erfasst, ob die Bewohnenden, die aufgrund des Depressionsverdachts eingeschlossen wurden, bereits vor der Pandemie an einer Depression erkrankt waren, oder ob dies eine Begleiterscheinung ebenjener war. Es ist denkbar, dass die Symptome bei diesen Befragten während der Pandemie eintraten.

Die untersuchte Stichprobe ist auf 9 Bewohnende begrenzt. Während die Bewohnenden bei Alter und Verweildauer eine heterogene Verteilung aufweisen, ist der Familienstand der 9 Befragten ähnlich (verwitwet, geschieden, ledig). Vier Teilnehmende haben zudem keine Kinder. Möglicherweise hatten die Bewohnenden bereits vor der Pandemie nur begrenzt soziale Kontakte, sodass die Kontakt- und Besuchsbeschränkungen für die Befragten weniger belastend sind. Es wurden keine Daten zu den sozialen Kontakten vor und während der Pandemie erfasst, somit kann hierzu keine Aussage getroffen werden.

Weiter muss bedacht werden, dass es sich bei der eingesetzten DIA‑S um ein Kurzscreening handelt und keine umfängliche Depressionsdiagnostik vorgenommen wurde. Die DIA‑S ersetzt keinesfalls eine ärztliche Diagnostik oder ein strukturiertes klinisches Interview und liefert lediglich einen Hinweis auf das Vorliegen einer depressiven Erkrankung auf Basis des Vorhandenseins depressiver Symptome.

Zu guter Letzt wurden keine Bewohnenden ohne Depressionsdiagnose oder -verdacht interviewt. Es kann daher kein Vergleich zwischen Bewohnenden mit und ohne depressiver Symptomatik bezüglich ihres Erlebens in der Pandemie gezogen werden. Es stellt sich die Frage, wie Bewohnende ohne depressive Symptome die Pandemie erleben, und ob und wie sich die Ergebnisse dieser beiden Gruppen unterscheiden würden.

Zu den Stärken der Studie zählt die Befragung einer in der Forschung eher unterrepräsentierten Zielgruppe. Der Zugang zu den Heimen und den Bewohnenden während der Pandemie und den geltenden Maßnahmen stellt ebenfalls eine Stärke der Studie dar.

## Fazit für die Praxis

- Die Erfahrungen und das Erleben der Heimbewohnenden mit depressiver Symptomatik zeigen sich in dieser beispiellosen Situation als sehr vielfältig und komplex. Ein individueller Zugang, z. B. in Form regelmäßiger vertrauter Gespräche zwischen Personal und Bewohnenden, zur Abklärung etwaiger Belastungen sowie unterstützende Angebote zur Vermeidung einer Verschlechterung der psychischen Verfassung sind wünschenswert.
- Es hat sich gezeigt, dass die Bewohnenden die viruseindämmenden Maßnahmen reflektieren, bewerten und einschätzen. Die Bewohnenden sollten bei der Implementierung einschränkender Maßnahmen in die zugrunde liegenden Entscheidungen einbezogen werden. Transparenz in Bezug auf die geltenden Einschränkungen sollte jederzeit gewährleistet sein.
- Die Bedürfnisse und Wünsche der Bewohnenden sollten auch in der Pandemie beachtet werden, und eine Abwägung zwischen Infektionsschutz und Erhalt der Lebensqualität sollte durch alle Beteiligten erfolgen.
